# The Coming Woman—Her Health

**Published:** 1892-02

**Authors:** S. H. Preston


### HALL'S JOURNAL OF HEALTH

TRUTH DEMANDS NO SACRIFICE; ERROR CAN MAKE NONE.

Vol. 39. FEBRUARY, 1892. No. 2.

### SPECIAL.

We very much desire the June, August, and September, 1888, numbers of our JOURNAL OF HEALTH, to complete a set of that year. Will some good friend send us either one or all of said numbers ?

---

### THE COMING WOMAN—HER HEALTH.

BY S. H. PRESTON.

Mankind may never look for a miraculous millennium—one ushered in with the pomp of Omnipotence and appalling phenomena. The same sun shall still shine on through countless centuries, and the eternal order of the universe continue undisturbed.

The means that have raised the race from savageism to civilization have been simple and natural. And all along the ages of ignorance, ignominy, and misery, the advancement of man has been astonishing. The same means are always meliorating his condition, and moving him onward and upward with mechanic might.

It is only when we turn back to the dark picture of the past—its brutal barbarisms, its bloody bigotries, its damning despotisms, that we can comprehend the vast advances that have been made. If so much has been already accomplished and by means so meagre, what may we expect from generations growing into general liberty and enlightenment ? The progress of the past is prophetic of a future for this planet far grander than poet or seer ever conceived.

The pivot of all progress, the foundation of all reform, is health. The medicated man, the weakly woman, cannot contribute to the welfare of the race. A sick person can do nothing well. And humanity is sick, its blood tainted with the transmitted diseases of a thousand generations.

Woman is the mother and the moulder of mankind. She is the director of human destiny. Her manifest mission is to bless and brighten our earth, and make it a better and more beautiful abode. She has always been the angelic agent of any advancement in this world.

*It was respect for woman that gave rise to chivalry, and the outcome of chivalry was civilization.*

The softening and refining influence of woman was felt in some sections of Europe, and the countries that vouchsafe to her sex its gentle supremacy became the conquerers of the world.

Civilization has gone forward or backward in exact accordance to the condition of woman. As far as her influence has been felt and favorably exerted, there has been great progress. When she has been suffered to remain in ignorance and slavery all progress has been arrested, or the race has relapsed into inertia and barbarism.

As woman is first in all progress, it follows she is first to be redeemed, and then to be the redeemer of the race. Were the women of the whole world what they should be, in health, wisdom, and womanly worth, the work of human redemption would be at once accomplished.

The first condition of a full development of woman is health. For generations she has been the victim of the vices of man—his drudge, or at best, the plaything of his passions. Her lot has been ignorance, neglect, and abuse, until unfitted for her duty as reproducer of the race.

Nature intended woman to be beautiful, healthy, and happy. But her multiplied miseries have prevented her from properly performing her maternal mission, impaired her physical functions, and poisoned the sources of new life.

The mothers of one generation mould the men and women of the next. A sick mother cannot produce healthy descendants. She is simply a transmitter of sickness to posterity. And the majority of mankind are born sick. So the chief causes of sickness are continued, and the tide of human infirmity flows forever onward.

In civilized life not one woman in five hundred is fitted for the office of motherhood, on account of complaints common to the sex. This may seem a startling statement, but it is amply attested by those peculiarly qualified by extensive professional experience to give an opinion.

And still, despite these facts, there are fore gleams of a more glorious future than ever imagined by man. There is dawning a new day of chivalry. The royalty of woman is being recognized as never before. She is being enthroned in the empire of the affections. The supremacy of her sex in the reproduction of a better breed of human beings is being acknowledged. As is the mother so will be her child, is now an accepted truth that will be made the means of infinite human improvement.

Creeds and customs are cracking. The chivalry of the future will consist in rescuing woman from the consequences of civilized wrongs. In enlightening her and freeing her from social fetters and outrages, we secure her health, elevate her character, and in so doing secure the welfare of posterity. It has been demonstrated that her influence is for the good of the race, when not perverted by the passion and ambition of man, and no one can doubt that her mission is finally to save and to bless. In giving her health we clear the fountain of life that flows onward through her offspring.

Woman's course in the future will present a striking contrast to her condition in the past. She will understand and obey the laws of life. She will be strong and intelligent, pure and beautiful, and will be the owner of her person and her property. The sexes shall stand squarely balanced on the scale of equality in every respect. Health and harmony and happiness will prevail, and sickness be something seldom, if ever, known.

"Lo! she is coming up the highway of the age,
A beautiful companion for philosopher and sage;
Her garments full of healing, her heart of heavenly grace,
And love surpassing splendor a shining in her face.

"O yes, she is coming, sweet mother of a race,
That shall love to do her honor and crown her in her place;
In councils of the nation and the holiest of homes.
Shall be heard the voice of woman when the coming woman comes."

